# Differences in the Dwell Time of Peripherally Inserted Central Catheters between Patients with Catheter Colonization and Those Developing Central Line-Associated Bloodstream Infection: A Single Centre Retrospective Cohort Study

**DOI:** 10.3390/antibiotics13070632

**Published:** 2024-07-08

**Authors:** Vassiliki C. Pitiriga, Elsa Campos, John Bakalis, George Saroglou, Athanasios Tsakris

**Affiliations:** 1 Department of Microbiology, Medical School, National and Kapodistrian University of Athens, 75 Mikras Asias Street, 11527 Athens, Greece; 2 Department of Internal Medicine, Metropolitan Hospital, 9 Ethnarchou Makariou Street, 18547 Athens, Greece

**Keywords:** central line-associated bloodstream infection (CLABSI), peripherally inserted central catheter (PICC), intravenous catheter, catheter colonization, multidrug-resistant pathogens

## Abstract

Substantial knowledge gaps exist concerning the varying durations of peripherally inserted central catheter (PICC) placements that lead to either central line-associated bloodstream infection (CLABSI) or catheter colonization. We aimed to compare PICCs dwell time between patients who developed CLABSIs due to multidrug-resistant microorganisms (MDROs) and patients with catheter colonization by MDROs. Data from 86 patients admitted consecutively to a tertiary-care hospital from 2017 to 2020 were retrospectively analyzed. The mean dwell time was 25.73 ± 16.19 days in the PICC-CLABSI group and 16.36 ± 10.28 days in the PICC-colonization group (*p* = 0.002). The mean dwell time was 17.38 ± 9.5 days in the PICC-MDRO group and 22.48 ± 15.64 days in the PICC-non-MDRO group (*p* = 0.005). Within the PICC-CLABSI group, the mean dwell time for CLABSIs caused by MDROs was 21.50 ± 12.31 days, compared to 27.73 ± 16.98 days for CLABSIs caused by non-MDROs (*p* = 0.417). Within the PICC-colonization group, the mean dwell time was 15.55 ± 7.73 days in PICCs colonized by MDROs and 16.92 ± 11.85 days in PICCs colonized by non-MDROs (*p* = 0.124). The findings of the present study suggest that CLABSIs caused by MDROs in PICCs are associated with a shorter mean catheter dwell time compared to those caused by non-MDROs, underscoring the importance of considering infections by MDROs when evaluating PICC dwell times.

## 1. Introduction

The use of peripherally inserted central catheters (PICCs) has become increasingly prevalent in modern medical practice. This popularity can be attributed to several factors, such as their ease of insertion, versatility in applications including medication administration and venous access, perceived safety, and cost-effectiveness compared to other central venous catheters [1,2]. Moreover, the establishment of nursing-led PICC teams has facilitated their widespread use across various healthcare settings [3].

However, despite the numerous advantages associated with PICCs compared to traditional central line catheters [4,5,6], they still carry a notable risk of central line-associated bloodstream infection (CLABSI), which can lead to serious complications and compromise patient outcomes [7]. PICCs can lead to CLABSIs due to several notable risk factors [8]. Improper aseptic techniques during insertion, such as insufficient skin disinfection or using non-sterile equipment, can introduce bacteria into the bloodstream [1]. Moreover, the catheter hub, if not adequately disinfected before each access, serves as a common entry point for pathogens. Frequent handling and manipulation of the catheter further increase contamination risks [1]. Patients with compromised immune systems or those requiring multiple catheter insertions are particularly vulnerable [9]. Additionally, incorrect catheter placement can cause mechanical irritation or thrombosis, promoting bacterial colonization [10]. Prolonged catheter use also provides more opportunities for infection, and improper maintenance, including infrequent dressing changes or failure to regularly inspect the insertion site for signs of infection, compounds these risks [11,12,13]. This risk stems from various factors, including the potential for biofilm formation on the catheter surface, which provides a favorable environment for bacterial colonization and subsequent bloodstream infection [14]. Moreover, with the emergence and spread of multidrug-resistant microorganisms (MDROs), the occurrence and management of CLABSIs has become increasingly challenging [15]. MDROs pose a significant threat to patient safety and healthcare systems globally, requiring close surveillance and strict infection control measures [16].

On the other hand, colonization of catheters without subsequent bloodstream infection is also a concern, as it can serve as a precursor to central line-associated bloodstream infections if the microorganisms gain access to the bloodstream. Previous studies suggest that the risk of colonization also increases with prolonged catheter dwell times [17,18]. In addition, our previous study comparing central venous catheters (CVCs) and PICCs [19] has shown that high rates of colonization by microorganisms, especially MDROs, arose later during catheterization in PICCs compared to CVCs. In this context, significant knowledge gaps remain regarding the duration of PICC placement leading to either infection or colonization by common pathogens, and most importantly by MDROs.

To address this critical gap in the knowledge, the present single-center retrospective study was conducted to compare the dwell time of PICCs between patients who developed CLABSIs due to MDROs and those with catheter colonization by MDROs, in an attempt to offer insights into the complex interplay between pathogen colonization, infection development, and antimicrobial resistance, and to highlight the importance of preventive measures in controlling MDRO infections within clinical environments.

## 2. Results

### 2.1. Participants Characteristics

A total of 86 patients with PICCs, 56 (65.1%) males and 30 (34.9%) females, with a mean age of 55.6 ± 21.1 years (ranging from 20 to 92 years), were included in the study. Out of these, 44 (51.2%) developed CLABSIs, while 42 (48.8%) had colonized catheters. The average duration of total participants’ catheterization was 20.94 ± 14.22 days (ranging from 3 to 72 days). Among all microorganisms isolated from PICCs, 26 (30.2%) were MDROs and 60 (69.8%) were non-MDROs. The detailed clinical characteristics of the patients are outlined in Table 1.

### 2.2. Comparison of the Dwell Times between PICCs Subgroups

The mean dwell time was 25.73 ± 16.19 days for the PICC-CLABSI subgroup and 16.36 ± 10.28 days for the PICC-colonization subgroup (*t* test, *p* = 0.002). Additionally, the mean dwell time for PICCs infected by non-MDROs was 22.48 ± 15.64 days whereas for PICCs with MDROs, it was 17.38 ± 9.5 days (*t* test, *p* = 0.005).

Within the PICC-CLABSI subgroup, the mean dwell time of catheters infected by MDR pathogens was 21.50 ± 12.31 days, while for catheters with non-MDROs, it was 27.73 ± 16.98 days (*t* test, *p* = 0.417). Within the PICC-colonization subgroup, the mean dwell time for catheters colonized by MDR pathogens was 15.55 ± 7.73 days, while for catheters with non-MDROs, it was 16.92 ± 11.85 days (*t* test, *p* = 0.124). Within the MDRO-PICC subgroup (*n* = 26), the mean PICC duration was 21.5 ± 12.31 days in CLABSIs events (*n* = 18) and 15.55 ± 7.73 days in colonization events (*n* = 8) (*t* test, *p* = 0.146). Within the non-MDRO-PICC subgroup (*n* = 60), the mean PICC duration was 26.7 ± 16.5 days in CLABSIs events (*n* = 34) and 16.9 ± 8.57 days in colonization events (*n* = 26) (*t* test, *p* = 0.011).

### 2.3. Identification and Distribution of MDROs

#### 2.3.1. Total PICCs

Among total PICCs, the most frequently isolated microorganisms were MDR Gram- negatives and fungi. More specifically, the most frequently isolated pathogen was MDR *Klebsiella pneumoniae* (*n* = 14, 16.3%) followed by MDR *Acinetobacter baumannii* (*n* = 10, 11.6%) *Candida albicans* (*n* = 9, 10.4%), and *Candida* non-*albicans* (*n* = 8, 9.3%).

#### 2.3.2. PICC-CLABSI Subgroup

No notable differences in prevalence were observed among the most frequent pathogens isolated from the PICC-CLABSI subgroup, including *Candida* non-*albicans*, MDR *K. pneumoniae*, *Staphylococcus aureus*, *Pseudomonas aeruginosa*, and *K. pneumoniae*, all sharing the same number of four (9.5%) isolations.

#### 2.3.3. PICC-Colonization Subgroup

In the PICC-colonization subgroup, the three most frequent pathogens were MDR *K. pneumoniae* (*n* = 10, 22.7%) which was the predominant microorganism isolated, followed by MDR *A. baumannii* (*n* = 8, 18.2%) and *C. albicans* (*n* = 6, 13.6%). Table 2. displays the microbial identification and distribution in the PICC-CLABSI and PICC-colonization subgroups.

**Figure 1:**
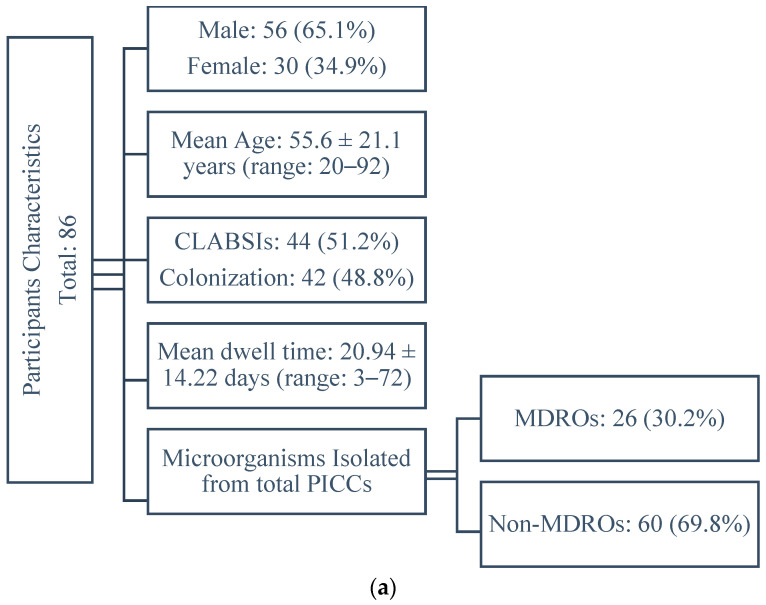

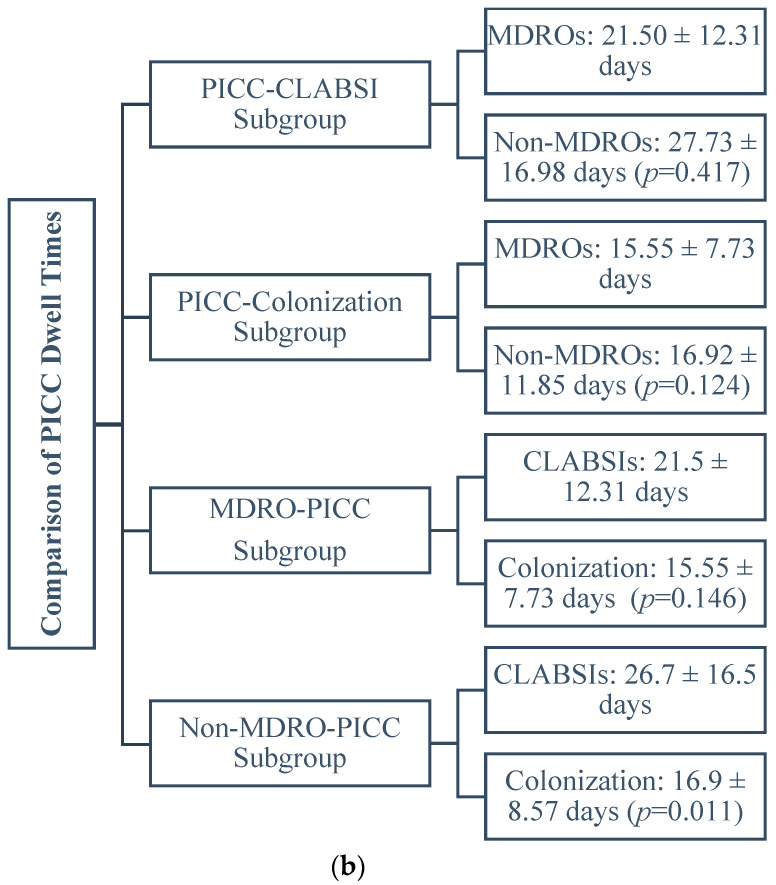

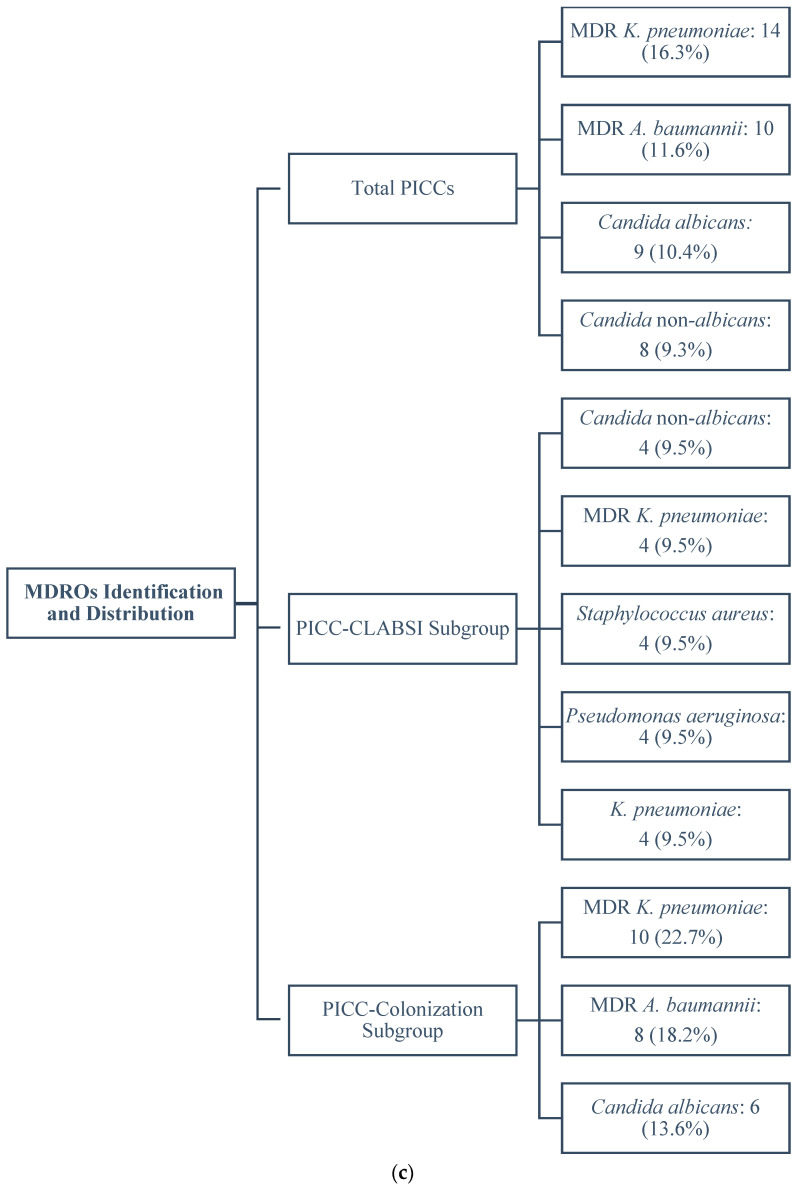
Flow diagram of study outcomes: (**a**) characteristics of the participants; (**b**) comparison of PICC dwell times; (**c**) MDRO identification and distribution. Abbreviations: *A. baumannii*, *Acinetobacter baumannii*; *K. pneumoniae*, *Klebsiella pneumoniae*; MDR, multidrug resistant; PICC, peripherally inserted central catheter; CLABSI, central line-associated bloodstream infection.

A flow diagram of the study outcomes is presented in Figure 1.

## 3. Discussion

To the authors’ knowledge, this is the first study to compare the impact of PICC placement duration on the development of CLABSI versus colonization by MDR pathogens. Our findings suggest that PICCs infected by MDROs had a shorter mean dwell time than those infected by non-MDROs. Of particular interest is the isolation of MDR pathogens in a substantial proportion of cases, accounting for approximately one-third of all isolated pathogens. This highlights the growing threat of antimicrobial resistance in healthcare settings and emphasizes the urgent need for tailored treatment approaches and antimicrobial stewardship initiatives.

The comparison of dwell times between PICCs that developed CLABSIs and colonized PICCs reveals notable differences between the two groups, since PICCs associated with CLABSIs exhibited a longer mean dwell time compared to those with colonization, suggesting an association between prolonged catheterization and the development of bloodstream infections. This finding aligns with previous research suggesting that a common cause of CLABSI involves initial colonization of central venous catheters, which may originate from the catheter hub or the surrounding skin [20,21].

Additionally, PICCs infected by MDR pathogens demonstrated a shorter mean dwell time compared to those infected by non-MDR pathogens. The same difference, although not at a statistically significant level, was exhibited when comparing the duration of catheter dwell time between PICCs infected with MDROs and those infected with non-MDR pathogens within PICC-CLABSIs and PICC-colonization subgroups. While numerous studies have examined the connection between antimicrobial resistance and catheter-related infections [22,23], research in this area typically centers on overall infection rates, pathogen profiles, and risk factors. However, there is currently a lack of data specifically comparing dwell times between catheter infections by MDROs and non-MDROs.

It is noteworthy that even though studies indicate lower infection rates for PICCs [24], nearly 50% of the patients in our sample presented with infections rather than colonization. We hypothesize that this trend could be attributed to the influx of a group of war-injured patients during this period, who presented with severe clinical symptoms and multiple severe wound infections and received prolonged antibiotic therapy, including combinations of antibiotics. PICCs were used for the treatment of this specific group.

The pathogen profile demonstrated by our study closely resembles previous reports from Greek hospitals, where MDR *A. baumannii* is frequently detected [25]. The emergence of MDROs is a significant concern in Greek hospitals, particularly among ICU patients [26]. According to annual data on antimicrobial resistance rates from our hospital’s diagnostic laboratories, more than 25% of nosocomial infections during the study period involved the three most commonly isolated MDR Gram-negative bacteria (*A. baumannii*, *K. pneumoniae*, *P. aeruginosa*), predominantly affecting ICU patients. Furthermore, a significant proportion of the cases in our study occurred in patients who underwent an extensive exposure to various antibiotic classes.

Our research demonstrated that the duration of PICC placement plays a crucial role in the acquisition of MDR pathogens. Unlike conventional pathogens, MDROs seem to colonize and cause infection relatively quickly after catheter insertion. One possible explanation for the shorter duration of catheter placement for acquiring MDR pathogens is that these organisms may have a heightened ability to adhere to catheter surfaces and establish biofilms compared to other pathogens. Biofilm production has emerged as a potential virulence factor in various bacteria, Gram-positives and Gram-negatives, as well as fungal species (such as *C. albicans* and *Candida* non-*albicans*), contributing to catheter colonization and catheter-related sepsis [27,28,29]. Especially in the case of *A. baumannii*, which emerged as the predominant MDR pathogen in our study, the chronicity and persistence of infections, coupled with its antibiotic resistance, are primarily associated with its ability to colonize and produce biofilms on diverse surfaces, including vascular catheters [30,31]. Biofilm-producing species have demonstrated elevated resistance to various classes of antibiotics. Notably, a significant correlation has been established between their biofilm formation capability and their level of antimicrobial resistance [32,33]. The genetic mechanism underlying increased horizontal gene transfer, observed in both resistant bacteria and biofilm-producing bacteria, may serve as the foundation for this correlation [34]. Other hypotheses for early infection by multidrug-resistant pathogens should also be considered at this point. Patient factors, such as multiple comorbidities or prolonged hospitalization, extensive antibiotic exposure, and the environment of intensive care units that can harbor multidrug-resistant organisms, may increase the risk of their introduction during PICC insertion or maintenance [35]. Most of these factors were present in our study population and mostly in the subgroup of the war-injured patients included in our study. The increased frequency of these risk factors in our study population also explains the relatively high mortality rate observed.

Our findings regarding the predominance of fungi in both PICC-colonization and PICC-CLABSI subgroups are concerning. *Candida* species represent a substantial portion of all isolates, pointing to the need for effective preventive measures and antifungal treatments. Despite their beneficial applications, venous catheters can increase the risk of fungal colonization, leading to local infections, venous inflammations, or, in rare cases [36,37], disseminated infections. Invasive candidiasis remains a major cause of mortality among hospitalized patients [38] and is the fourth most common cause of hospital-acquired bloodstream infections in the United States [39]. While the rising prevalence of *Candida* bloodstream infections is largely linked to the use of CVCs [40,41], our findings also indicate a significant presence of these infections associated with PICCs.

## 4. Materials and Methods

### 4.1. Study Design

We performed a retrospective analysis of clinical data from critically ill patients consecutively admitted at Metropolitan Hospital, a tertiary-care private hospital in Athens, Greece, from May 2017 to May 2020. Data from patients who had PICCs during that period and developed either CLABSIs or catheter colonization were selected for analysis. Patients who had more than one PICC in place simultaneously (concurrent PICCs) were excluded from the study analysis. After catheter insertion, a checklist form was used, which included the patient’s diagnosis, operator’s name, chosen site, insertion and removal dates, date of ICU discharge or death, use of mechanical ventilation, arterial catheters, parenteral nutrition, vasopressor support, and daily clinical assessments (such as induration, discharge, erythema, and tenderness) to monitor potential catheter-related infections. The operator responsible for catheter insertion initially recorded the data, while nursing staff continued data entry on subsequent days. Additionally, infection control nurses monitored data collection 3–4 times per week.

Study data were retrospectively gathered from three distinct sources: the electronic clinical records provided demographic and clinical information related to patient admission and progress; the clinical laboratory; and the hospital infection control team database.

The definitions used in the study were as follows: (a) *Peripherally inserted central catheters (PICCs)* were categorized as catheters inserted into the basilic, cephalic, or brachial veins of the upper extremities, with tips terminating in the superior vena cava or right atrium; (b) *Multidrug-resistant microorganisms (MDROs)* were identified as microorganism species exhibiting antimicrobial resistance to at least one antimicrobial drug in three or more antimicrobial categories. This definition encompassed both Gram-positive and Gram-negative bacteria; (c) *Catheter-associated bloodstream infection (CLABSI)* was defined as a laboratory-confirmed bloodstream infection (a positive blood culture with no other apparent source of infection) occurring in the presence of a CVC or within 48 h of CVC removal; and (d) *Catheter colonization* was considered the presence by a semi-quantitative culture of ≥15 CFU of at least a single organism per catheter, according to Maki [42].

Ethical approval for this observational study was obtained from the hospital’s institutional review board.

### 4.2. Catheter Care

Care bundles implemented in our hospital are in accordance with guidelines on catheter-related infection prevention recommended by CDC and other health organizations (IDSA, etc.) [20]. Highly skilled nursing personnel proficient in all facets of catheter care maintained the standardized catheter care protocols. To reduce the risk of dressing contamination, thorough visualization of all insertion sites was conducted. Dressings were replaced every few days or more frequently as deemed clinically necessary. Nursing staff utilized iodine solution to cleanse the skin site and catheter hub during dressing changes and also replaced the intravenous accessory tubing. Moreover, strict adherence to sterile insertion techniques was observed by the nursing staff. Catheters were removed in the following circumstances: (a) suspicion of infection, (b) when no longer needed.

### 4.3. Culture Techniques

All catheters were subjected to pathogen examination either routinely after removal or if infection was suspected. The process involved disinfecting the skin around the catheter entry site followed by excising the proximal 4–5 cm portion of the tip using sterile scissors. The excised specimen was then placed in a sterile container and promptly transported to the microbiology laboratory within 15 min at room temperature. Analysis of both the intradermal and intravascular portions of the catheter was conducted using the semi-quantitative culture technique described by Maki et al. [42]. According to this technique, a positive catheter tip culture was defined as the presence of ≥15 colony-forming units (CFUs) of any microorganism. Blood cultures were incubated in aerobic and anaerobic broth media using the Becton Dickinson BACTEC system. Identification of isolates and determination of antimicrobial resistance patterns were performed using the VITEK^®^2 Automated Compact System. Additionally, an E-test was conducted to confirm resistance phenotypes reported by the VITEK System, following standard laboratory procedures. The Clinical and Laboratory Standards Institute (CLSI) criteria and breakpoints were followed for interpretation of antimicrobial susceptibility tests.

### 4.4. Statistical Analysis

Descriptive analyses were conducted to characterize the patient population. Qualitative variables are reported as counts and percentages, while quantitative variables are expressed as mean values with standard deviations (±). Comparisons among the groups were performed using an independent samples *t*-test. A *p*-value of less than 0.05 was considered statistically significant. Statistical analysis was performed using the SPSS package (version 28.0).

## 5. Conclusions

Our findings that the duration of catheter placement for acquiring MDR pathogens is shorter than that for acquiring non-MDR pathogens in PICCs highlights the need for proactive infection control measures and ongoing surveillance to minimize the risk of catheter-related infections. They also underscore the urgency of implementing stringent infection prevention and control measures from the moment of PICC insertion. Healthcare providers should balance the need for long-term intravenous access with the associated infection risks, especially those related to MDROs. To mitigate these risks effectively, they should implement strategies such as regular catheter site care, timely removal of catheters when no longer needed, and adherence to evidence-based insertion and maintenance practices. Moreover, while *Candida* bloodstream infections are primarily associated with the use of CVCs, our study suggests that there is also a notable presence of these infections linked to PICCs.

## Figures and Tables

**Table 1:** Study population’s demographic and clinical characteristics upon admission and during hospital stay.

Patients’ Characteristics (*n* = 86)	N (%)
Respiratory disorders	23 (26.7)
Trauma	26 (30.2)
Diabetes mellitus	20 (23.2)
Hypertension	48 (55.8)
Cerebrovascular diseases	40 (46.5)
Gastrointestinal disease	19 (22)
Kidney disease	18 (20.9)
Oncological Disorders	20 (23.2)
Cardiovascular disease	25 (29.0)
Immune deficiency/suppression	45 (52.3)
***During hospital stay***	
ICU admission	50 (58.1)
Total parenteral nutrition	35 (40.7)
Mechanical ventilation	31 (36.0)
Prolonged hospitalization (>1 month)	45 (52.3)
Death	15 (17.4)
Sepsis	15 (17.4)
APACHE II at inclusion (mean ± SD)	12.9 ± 7.5

Abbreviations: SD, Standard Deviation; *n*, N number; ICU, intensive care unit.

**Table 2:** Pathogen distribution among PICC CLABSIs and colonization subgroups.

Microorganisms	Clabsi No (%)	Colonization No (%)
**Fungi**		
*Candida albicans*	3 (7.1)	6 (13.6)
*Candida non-albicans*	4 (9.5)	4 (9.1)
Other fungi	2 (4.8)	-
**Gram-negatives**		
*Enterobacter cloacae*	3 (7.1)	-
*Klebsiella pneumoniae*	4 (9.5)	-
*Pseudomonas aeruginosa*	4 (9.5)	2 (4.5)
*Proteus mirabilis*	1 (2.4)	2 (4.5)
*Serratia marcescens*	2 (4.8)	-
MDR *Acinetobacter baumanni*	2 (4.8)	8 (18.2)
MDR *Klebsiella pneumoniae*	4 (9.5)	10 (22.7)
MDR *Pseudomonas aeruginosa*	2 (4.8)	-
**Gram-positives**		
*Staphylococcus aureus*	4 (9.5)	-
MRSA	-	2 (4.5)
*Staphylococcus haemolytic*	2 (4.8)	2 (4.5)
*Streptococcus salivarious*	-	2 (4.5)
*Enterococcus faecalis*	2 (4.8)	-
*Enterococcus faecium*	-	4 (9.1)
CnS	3 (7.1)	2 (4.5)
**Total**	**42**	**44**

Abbreviations: CnS, coagulase-negative *Staphylococcus*; MRSA, Methicillin-resistant *Staphylococcus aureus*; MDR, multidrug-resistant.

## Data Availability

Data are contained within the article.
